# Conformality limits of 2D WS_2_ on 3D nanostructures†

**DOI:** 10.1039/d5nr01013f

**Published:** 2025-07-01

**Authors:** Jeff J. P. M. Schulpen, Saravana B. Basuvalingam, Marcel A. Verheijen, Ageeth A. Bol

**Affiliations:** a Department of Applied Physics and Science Education, Eindhoven University of Technology Eindhoven 5600 MB the Netherlands aabol@umich.edu; b Eurofins Materials Science BV High Tech Campus Eindhoven 5656 AE the Netherlands; c Department of Chemistry, University of Michigan Ann Arbor MI 48109 USA

## Abstract

3D nanostructures are a vital part of various applications envisaged for two-dimensional transition metal dichalcogenides (2D TMDs), such as nanoelectronics and catalysis. However, achieving conformal deposition of 2D TMD films on 3D nanostructures is challenging due to the requirement for bending the basal planes of the 2D TMDs. Here, the limits of conformality of 2D WS_2_ deposited by atomic layer deposition on SiO_2_ 3D nanostructures are investigated through cross-sectional transmission electron microscopy imaging. A minimum radius of curvature of 4 nm is identified above which basal plane conformality is almost always observed, while for smaller radii conformality is only observed in approximately half of the cases. We show that the observed tipping point agrees with the balance between the adhesion and stiffness forces, which allows for the estimation of the critical radius of curvature for other 2D TMDs and substrates. These results provide guidelines for the design of 3D nanostructured devices and substrates on which conformality of 2D materials is desired.

## Abstract 2

A critical radius of curvature of 4 nm for conformal deposition of WS_2_ on 3D nanostructures was identified, offering design guidelines for integrating 2D transition metal dichalcogenides in advanced nanoelectronic and catalytic applications.

## Graphical abstract

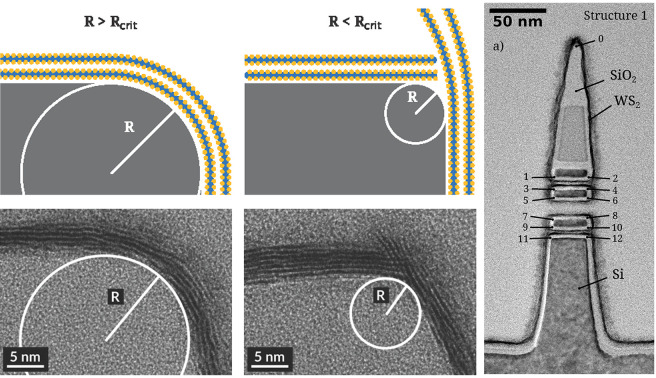

## A. Introduction

The attractive physical and electronic properties of two-dimensional transition metal dichalcogenides (2D TMDs) such as high carrier mobility, strong exciton binding and the ability to be thinned down to a single monolayer without deteriorating their properties, make this class of materials of high interest for various applications in sensing, (opto)electronics and more. Specific applications require conformal coating of 2D TMD layers on nanostructures with surfaces of high curvature. For example, nanostructured substrates are becoming ever more important in the semiconductor industry as three-dimensional transistor structures have become the norm in *e.g.* finFETs. This trend towards complex nanostructured transistors is only expected to continue with the imminent large-scale adoption of gate-all-around FETs (also referred to as nanosheet FETs), likely followed by forksheet FETs and possibly stacked complementary FETs.^1^ In addition, bending of the 2D TMD basal planes is essential in for example multi-bridge channel FETs.^2^ Integration of 2D TMDs in future transistor architectures is expected to further boost transistor performance, making it of specific interest to study the compatibility of these materials with such high-curvature nanostructured substrates. A second example is the coating of nanoparticles for energy applications, which have radii on the order of 10 nm, and may locally have even more extreme curvature radii on the order of 1 nm at facet edges and corners.^3^ Similarly, single-walled carbon nanotubes have curvature radii as low as 1 nm.^4^ The conformality of the basal planes of a 2D TMD on such structures is not trivial since this requires bending of the basal planes to conform to the structure, which requires an amount of energy dictated by the bending stiffness of the 2D TMD. Nevertheless, high curvature of 2D TMD basal planes can be obtained. For example, TMD nanoscrolls, which are obtained by rolling up flakes of MoS_2_, WS_2_, MoSe_2_ and WSe_2_, typically have inner radii ranging from to 5 to 20 nm.^4^ Furthermore, nanotubes of MoS_2_ are observed with inner radii down to 4 nm. Similarly, WS_2_ nanotubes are observed with an inner radius as small as 3 nm.^5^ In the presence of a substrate, adhesion of the 2D TMD to the substrate is an additional effect that determines the maximum curvature of 2D TMDs. Specifically, the balance between the stiffness of the 2D TMD and its adhesion to the substrate determines whether the TMD nanosheets will remain conformal to a substrate or peel off. In one study, growth of conformal MoS_2_ by CVD onto boron nitride nanotubes (BNNTs) was only observed for BNNT radii larger than approximately 2.5 nm.^6^ In another investigation, growth of conformal MoS_2_ monolayers by CVD on carbon nanotubes was only observed for MoS_2_ radii larger than 2 nm.^7^ The low yield of this process (<1% MoS_2_ coverage across all nanotubes) was attributed to the fact that most CNTs had a radius less than 1.5 nm before MoS_2_ deposition. By coating the CNTs with h-BN before MoS_2_, their radii could be increased to >1.5 nm. However, the MoS_2_ yield still did not approach 100%, instead seeming to level off around 10%. These results suggest there may be different regimes of basal plane conformality on highly curved substrates: a “high” curvature regime where basal plane conformality is never observed, a “low” curvature regime where basal plane conformality is always observed, and perhaps a “medium” curvature regime where basal plane conformality is inconsistent. Studies of CVD-grown MoS_2_ and WS_2_ on Co_9_S_8_ nanoparticles demonstrate basal plane conformality even on facet edges which have a local radius of curvature <1 nm.^3,8^ However, defectivity and formation of grain boundaries in the TMD film is observed at these sharp edges. Furthermore, the growth of the MoS_2_ and WS_2_ onto Co_9_S_8_ was observed to be epitaxial, which may improve the TMD conformality on these specific substrates compared to the general case of non-epitaxial substrates. This brief survey of the recent literature on conformality of 2D TMDs on nanostructures indicates that the limits beyond which the conformality breaks down are not clearly understood.

Atomic layer deposition (ALD) is known for its exceptional film thickness uniformity, even on complex nanostructured substrate. This advantage results from the use of alternating self-limiting vapor-surface reactions, which lead to surface-controlled (rather than flux-controlled) film growth. Since this thickness uniformity is often an essential requirement for deposition on nanostructured substrates, ALD is a highly suitable deposition method for such applications. So far, basal plane conformality of ALD-deposited TMDs has been demonstrated down to a radius of curvature of 10 nm.^9^ Although this radius is not as small as reported for CVD-grown TMDs as mentioned earlier, to the best of our knowledge no attempts have been made to coat nanostructures with smaller radii of curvature by ALD. In this work, we investigate the limits of the conformality of an ALD grown 2D transition metal dichalcogenide on high-curvature nanostructures. As a model material we grow WS_2_, which is a semiconducting TMD that has received widespread attention for use as a channel material in transistors based on 2D materials.^10^ As nanostructures, we employ gate-all-around field effect transistor (GAA-FET) structures, which provide the kind of curved SiO_2_ surfaces that are currently industrially relevant for the semiconductor industry. Beyond their industrial relevance, these substrates also constitute a general platform on which the curvature-dependent conformality of 2D materials can be studied to inform the growth on other highly curved substrates.

## B. Results

### Structures and curvatures

**Fig. 1:**
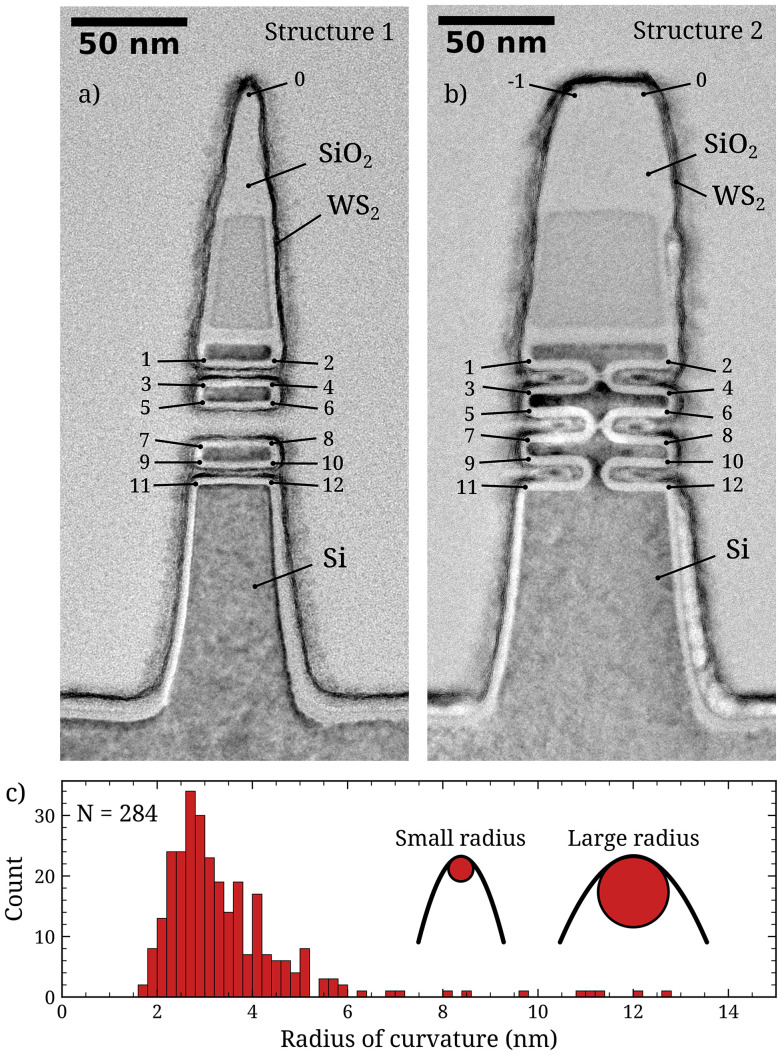
(a and b) The two 3D nanostructures used in this work. The places of high curvature where basal plane conformality was evaluated are indicated and numbered. (c) Histogram of the measured radii of curvature. The inset illustrates the concept of radius of curvature: a smaller radius corresponds to a sharper corner.

As test structures, two types of gate-all-around FET structures were used, which are shown in Fig. 1a and b. The fins of type 1 (Fig. 1a) are etched completely through in 3 locations along its height, such that they have 13 places of positive curvature: one at the top of the fin (spot 0) and 12 along the sides (spot 1–12). The fins of type 2 (Fig. 1b) are broader and are etched incompletely, such that they have 14 places of positive curvature: 2 at the top of the fin (spot −1 and 0) and 12 along the sides (spot 1–12). There are also places of negative curvature at the base of both structures, and internally in the structures of type 2 at the endpoints of the etch. However, in the present study we disregard the places of negative curvature for three reasons. Firstly, delamination at the base of the fins originating from preparation of the TEM samples hampers quantification. Secondly, crystalline WS_2_ could not be observed at the internal locations with negative curvature, which is attributed to the lack of exposure to reactive plasma species of these internal regions during the ALD process. Finally, the conformality of the WS_2_ basal planes in locations with negative curvature is challenging to classify based on cross-sectional TEM data. While in locations with positive curvature non-conformality often results in “overhanging” WS_2_ planes (discussed below), this does not happen in locations with negative curvature. Instead, inability of the 2D TMD to conform to negative curvature may result in the formation of defects in the form of grain boundaries. As the ALD-grown WS_2_ inherently already has small grain sizes on the order of 10 nm, such an additional effect will be hard to observe from cross-sectional TEM imaging. Hence, in this study we consider only the locations with positive curvature on the nanostructures. In total, 284 locations of positive curvature were studied from 14 structures of type 1, and 9 structures of type 2. The histogram of the radii of curvature of all these places is shown in Fig. 1c. The associated curvature determination can be found in the ESI.† The radii of curvature range from 1.6 to 12.7 nm with a median of 3.1 nm. Most radii (93%) are between 2 and 6 nm.

### Classifying conformality of deposited WS_2_

**Fig. 2:**
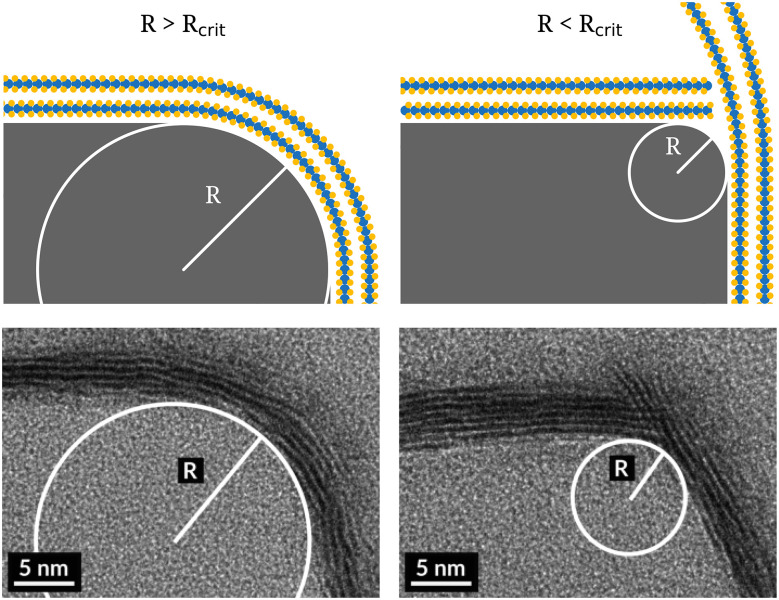
Schematic and real examples of conformality and nonconformality of ALD-grown WS_2_.

The conformality of the PEALD-grown WS_2_ at all 284 edges was classified as either being conformal, non-conformal or indeterminable based on visual inspection. A list of all edges and their classifications can be found in the ESI.† The classification “conformal” was given when the basal planes nearest to the substrate were seen to follow the curvature of the substrate. An example is given in Fig. 2. Since the deposited film thickness was approximately 8 basal planes in this study, it often occurred that the top few planes did not conform to the substrate, while the ones at the bottom did. This is similar to the effect seen on planar substrates where with increasing thickness more planes start to grow perpendicularly to the substrate.^11,12^ Hence, such non-conformality is not necessarily related to the curvature of the substrate, and in these cases the film was still classified as conformal as long as the basal planes at the bottom conformed to the nanostructure. On the other hand, the classification “non-conformal” was given when these bottom basal planes did not conform to the substrate edge structure, which could often be identified visually as “overshooting” basal planes beyond an edge, as illustrated in Fig. 2. In such a situation, the basal planes extended outward off the substrate rather than following the curvature of the substrate. Lastly, the classification “indeterminable” was used for edges where the conformality of the WS_2_ basal planes could not be verified, for example because no basal planes were visible. An example of an edge that received the classification ‘indeterminable’ is given in the ESI.†

### Results of classification

**Fig. 3:**
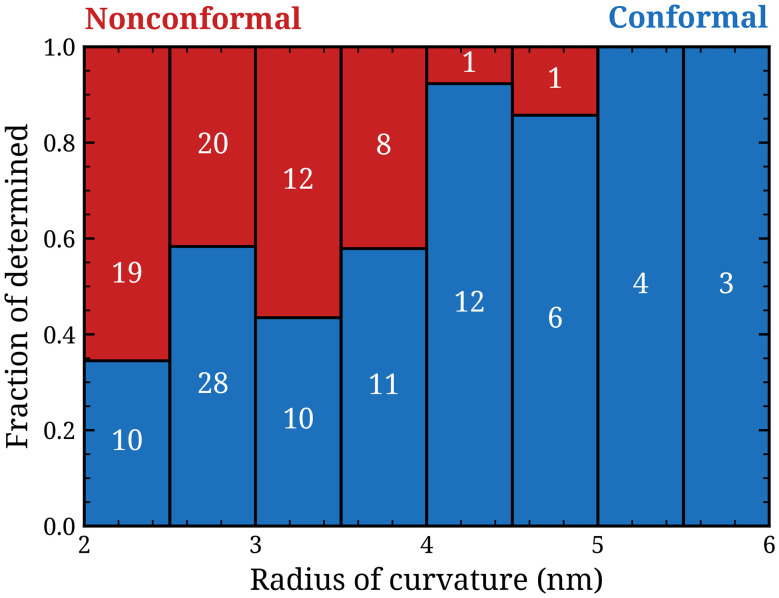
Results of the classification of the WS_2_ on the nanostructure edges as either conformal or nonconformal, separated by radius of curvature.

Out of the 284 locations with positive curvature, 94 were classified as conformal, 66 as non-conformal and 124 as indeterminable. Over 70% of the instances of indeterminable conformality occurred on “down-facing” corners, *i.e.* those labeled 1, 2, 5, 6, 9 and 10 in Fig. 1a. It is expected that such down-facing corners received less ion bombardment during the H_2_S plasma step of the ALD process due to the directionality of the ions perpendicular to the substrate surface. This may lead to a lower crystallinity of the WS_2_ at these down-facing corners, making the evaluation of the conformality at these corners difficult. The indeterminable fraction did not depend on the radius of curvature and is therefore not further considered. Fig. 3 shows the relative amount of conformal and non-conformal growth as a function of the radius of curvature. For small radii of curvature, the fractions conformal and nonconformal are equal: between radii of 2 and 4 nm, 59 edges were classified conformal and 59 edges were classified non-conformal. For larger radii of curvature there was a strong tendency towards conformality: for radii between 4 and 6 nm only 2 cases were classified as non-conformal while 25 were classified as conformal. The balance between conformal and nonconformal growth clearly depends on the radius of curvature. Below a curvature radius of 4 nm nonconformality was common, while it was virtually absent for edges with a radius of curvature larger than 4 nm.

## C. Discussion

### Adhesion-stiffness energy balance

The basal plane conformality of the deposited film is governed by the competition between two effects: adhesion of the TMD to the substrate surface and the bending stiffness of the TMD film. When adhesion dominates, conformality is expected, while dominating bending stiffness should lead to non-conformality. The energy associated with the bending of the 2D TMD layer (*E*_bending_) scales quadratically with curvature:^13^

1 *E*_bending_ = *Bκ*^2^*A* = (*B*/*R*^2^)*A*

where *B* is the bending stiffness (units eV), *κ* is the curvature, *R* is the radius of curvature and *A* is the area. The energy associated with the adhesion of the TMD to the substrate (*E*_adhesion_) can be expressed as:

2 *E*_adhesion_ = *ΓA*

where *Γ* is the areal energy density of adhesion and *A* is the area. Then, adhesion and stiffness are in equilibrium when:

3 (*B*/*R*^2^)*A* = *ΓA*

which is satisfied at a critical radius of curvature (*R*_crit_):

4 *R*_crit_ = √(*B*/*Γ*)

For weak curvature *R* > *R*_crit_, adhesion dominates, and conformal growth is expected. For large curvature (*R* > *R*_crit_), stiffness dominates, and non-conformal growth is expected. From our experimental results (Fig. 3), it appears that *R*_crit_ for WS_2_ on SiO_2_ is approximately 4 nm. We now estimate the theoretically expected value for *R*_crit_ based on literature values for the bending stiffness of WS_2_ and its adhesion energy on SiO_2_. The elastic properties of MoS_2_ and WS_2_ have previously been reported to be very similar.^14^ Recently Yu *et al.* have accurately determined the bending stiffness of exfoliated MoS_2_ in good agreement with theoretical results.^13^ They find a bending stiffness of 10.15 ± 1.4 eV per layer for bilayer MoS_2_. For aligned (2H phase) atomic layers, the bending stiffness was found to depend on the curvature, while for misaligned (*i.e.* twisted) layers the stiffness was independent of curvature. Since the alignment of lattice planes of ALD-grown 2D TMDs is generally not perfect, we adopt the stiffness value of 10.15 ± 1.4 eV per atomic layer, independent of curvature, and assume a linear relationship between the number of layers and bending stiffness.^13^ Measurements of adhesion energy are challenging due to the difficulty of excluding surface contaminants which affect the adhesion behavior. Again, little data on WS_2_ is available. However, it has been shown through *ab initio* calculations that the energy required for exfoliation of WS_2_ is very similar to that of MoS_2_.^15^ Since the exfoliation energy is closely linked to the (self)adhesion, we may assume the adhesion energies of WS_2_ and MoS_2_ on SiO_2_ are similar too. Various studies report the adhesion energy of exfoliated MoS_2_ on SiO_2_. One study using shape analysis of spontaneously formed blisters in MoS_2_ film filled with surface contaminants (assumed to be water and an unknown quantity of hydrocarbons) yields a value of the areal adhesion energy density of 82 ± 1 mJ m^−2^.^16^ Another study uses the same methodology to study MoS_2_ blisters formed by gold nanoparticle intercalation, yielding a value of ∼482 mJ m^−2^.^17^ Dual cantilever beam fracture analysis is another method to measure film adhesion, with one study reporting a value of 2200 ± 100 mJ m^−2^ for MoS_2_ on SiO_2_.^18^ Lastly, a shape analysis study of an open-ended MoS_2_ wrinkle in air yielded a value of 170 ± 33 mJ m^−2^,^19^ and a similar study on gas-pressurized MoS_2_ blisters yielded a value of 220 ± 35 mJ m^−2^.^20^ As these last two studies have minimal influence of surface contaminants and yield mutually consistent results, we average these results and adopt a value of 200 ± 50 mJ m^−2^ for the surface adhesion of WS_2_ on SiO_2_. Using these literature values for the adhesion and stiffness of WS_2_, we can calculate the critical radius of curvature to be 2.8 ± 0.7 nm. This number is consistent with our experimentally found value of 4 ± 0.5 nm, indicating that the transition in basal plane conformality can indeed be understood by competing effects of adhesion and stiffness. These results also demonstrate that the ratio of bending stiffness and adhesion (and thus the conformality) of ALD-grown WS_2_ does not deviate significantly from its value for CVD-grown and exfoliated TMDs, despite differences in grain sizes. The grain sizes of ALD-grown TMDs are typically on the order of 10 nm and are therefore much smaller than the micron-sized grains which can be achieved by CVD and exfoliation. For graphene the effect of grain sizes and defects on the stiffness was also found to be small, with one study finding similar stiffnesses for films with grain sizes of 1–5 μm and 50–200 μm.^21^ Another graphene study showed its stiffness was not affected by sp^3^ type point defects even up to a defect spacing of 5 nm, although vacancy defects did decrease the stiffness and strength of the graphene.^22^ Lastly, it would be interesting to see how the results for locations with positive curvature derived in this work extend to locations with negative curvature. Recently, extreme bendability of TMDs on negative curvatures was demonstrated.^23^ It may be the case that smaller radii of curvature are tolerable when the curvature is negative instead of positive. However, proper statistics on such negative curvature structures are needed to confirm this hypothesis.

## D. Methods

GAA-FET nanostructures were graciously supplied by TSMC and were made using standard deposition, lithography and etch procedures. In all cases the outer surface of the 3D nanostructures was SiO_2_. WS_2_ films were grown directly onto the 3D nanostructures using the previously published plasma-enhanced ALD process^12^ in an Oxford Instruments FlexAL reactor using W(NtBu)_2_(NMe_2_)_2_ as the tungsten precursor and a 200 W remote ICP plasma with a 10 : 40 sccm H_2_S : Ar gas mixture as co-reactant, at a substrate table temperature of 450 °C. A total of 50 cycles was performed, yielding a WS_2_ thickness of approximately 5 nm, corresponding to ∼8 WS_2_ layers. TEM studies have been performed using a probe-corrected JEOL ARM 200F, operated at 200 kV. Curvature radii were estimated manually from the cross-sectional TEM images using ImageJ software.

## E. Conclusions

We have conformally deposited WS_2_ nanolayers on 3D SiO_2_ nanostructures by atomic layer deposition and quantified the conformality of the WS_2_ to the substrate structure as a function of the local substrate curvature. We found that for curvature radii > 4 ± 0.5 nm, conformal growth is obtained in virtually all cases. On the other hand, for lower curvature radii down to 2 nm, conformal growth was observed only in approximately half of cases. To understand this transition, we formulated a balance equation of the stiffness and adhesion of the 2D TMD on SiO_2_. This calculation yielded a critical radius of curvature of 2.8 ± 0.7 nm at which adhesion and stiffness of the 2D TMD are in equilibrium. For smaller radii of curvature, stiffness of the 2D TMD dominated and non-conformal growth of the 2D TMD was expected. For larger radii curvature, adhesion dominates, and conformal growth was expected. The calculated critical radius of 2.8 ± 0.7 nm was in reasonable agreement with the experimentally found value of 4 ± 0.5 nm. Our method of calculation allows such minimal radii of curvature to be determined for other TMD/substrate systems if adhesion energies and bending stiffness of the TMD are known. These results can inform the design of 3D nanostructures on which conformal growth of a TMD film is required.

## Author contributions

J. J. P. M. S., M. A. V. and A. A. B.: conceptualization, J. J. P. M. S., S. B. B. and M. A. V.: data curation and investigation, J. J. P. M. S.: formal analysis, A. A. B.: funding, project administration, A. A. B. and M. A. V.: supervision, J. J. P. M. S.: writing – original draft, S. B. B., M. A. V. and A. A. B.: writing – review and editing.

## Conflicts of interest

There are no conflicts to declare.

## Supplementary Material

NR-017-D5NR01013F-s001

## Data Availability

The data supporting this article have been included as part of the ESI.†
