# Supplementary material for: Conformality limits of 2D WS2 on 3D nanostructures
Source: Nanoscale. 2025 Jul 1;17(28):16922–7. doi: 10.1039/d5nr01013f (PMC12230646; doi:10.1039/d5nr01013f)
Supplement: NR-017-D5NR01013F-s001 [file NR-017-D5NR01013F-s001.pdf]

Supplementary Information (SI) for Nanoscale.
This journal is © The Royal Society of Chemistry 2025

Supplemental Information

Conformality limits of 2D WS_2 on 3D nanostructures

Jeff J. P. M. Schulpen[1], S. Basuvalingam[1], Marcel A. Verheijen[1,2], Ageeth A. Bol[1,3]

[1]*Department of Applied Physics and Science Education, Eindhoven University of Technology, Eindhoven 5600 MB, the Netherlands*

[2]*Eurofins Materials Science BV, High Tech Campus, Eindhoven 5656 AE, the Netherlands*

[3]*Department of Chemistry, University of Michigan, Ann Arbor, MI 48109, USA*

Classifications of curvature radius and basal plane conformality per corner (maybe = undeterminable). All TEM data that supports this work can be found here: https://doi.org/10.7302/6z5j-fk89

fin	corner	type	radius	conformal
A1	0	top	1.81	maybe
A1	1	down	2.79	yes
A1	2	down	4.17	yes
A1	3	up	2.70	yes
A1	4	up	2.36	no
A1	5	down	1.62	maybe
A1	6	down	2.72	maybe
A1	7	up	2.93	yes
A1	8	up	1.91	no
A1	9	down	2.15	yes
A1	10	down	3.44	yes
A1	11	up	1.85	maybe
A1	12	up	2.95	yes
A2	-1	top	12.72	yes
A2	0	top	5.12	maybe
B1	0	top	2.08	no
B1	1	down	2.68	no
B1	2	down	5.04	maybe
B1	3	up	1.81	yes
B1	4	up	2.53	maybe
B1	5	down	1.62	yes
B1	6	down	3.59	maybe
B1	7	up	2.06	no
B1	8	up	2.36	yes
B1	9	down	2.06	maybe
B1	10	down	3.51	yes
B1	11	up	2.93	yes
B1	12	up	2.61	yes
B2	-1	top	12.09	yes
B2	0	top	4.72	maybe
B2	1	down	5.59	maybe
B2	2	down	5.89	maybe
B2	3	up	5.77	yes
B2	4	up	5.09	maybe
B2	5	up	4.44	maybe
B2	6	down	4.04	maybe
B2	7	up	4.06	yes
B2	8	up	4.76	no
B2	9	down	3.75	maybe
B2	10	down	4.65	maybe
B2	11	up	5.14	yes
B2	12	up	2.93	no
C1	0	top	2.93	yes
C1	1	down	3.10	yes
C1	2	down	4.85	yes
C1	3	up	2.61	yes
C1	4	up	2.36	no
C1	5	down	2.08	maybe
C1	6	down	3.91	yes
C1	7	up	2.51	yes
C1	8	up	2.85	yes
C1	9	down	2.63	no
C1	10	down	2.55	maybe
C1	11	up	2.51	yes
C1	12	up	2.44	no
C2	-1	top	11.01	yes
C2	0	top	8.59	yes
C2	1	down	3.68	maybe
C2	2	down	5.07	maybe
C2	3	up	3.87	yes
C2	4	up	4.95	maybe
C2	5	down	3.36	no
C2	6	down	4.36	maybe
C2	7	up	4.39	yes
C2	8	up	4.15	maybe
C2	9	down	4.18	maybe
C2	10	down	4.18	maybe
C2	11	up	4.15	maybe
C2	12	up	2.09	no
D1	0	top	2.91	yes
D1	1	down	3.15	no
D1	2	down	5.06	yes
D1	3	up	2.47	yes
D1	4	up	2.79	yes
D1	5	down	2.81	maybe
D1	6	down	4.04	yes
D1	7	up	2.72	yes
D1	8	up	2.95	yes
D1	9	down	2.32	maybe
D1	10	down	3.51	maybe
D1	11	up	2.49	yes
D1	12	up	2.63	yes
D2	-1	top	11.38	yes
D2	0	top	9.79	maybe
D2	1	down	2.60	no
D2	2	down	4.01	maybe
D2	3	up	1.85	no
D2	4	up	2.82	no
D2	5	down	2.46	maybe
D2	6	down	2.75	maybe
D2	7	up	3.07	yes
D2	8	up	2.63	no
D2	9	down	2.25	maybe
D2	10	down	3.07	maybe
D2	11	up	2.46	maybe
D2	12	up	2.39	maybe
E1	0	top	3.57	yes
E1	1	down	2.95	yes
E1	2	down	3.64	no
E1	3	up	2.55	no
E1	4	up	2.32	no
E1	5	down	3.06	maybe
E1	6	down	3.17	maybe
E1	7	up	2.72	no
E1	8	up	2.57	no
E1	9	down	3.27	yes
E1	10	down	3.76	maybe
E1	11	up	2.66	no
E1	12	up	2.64	yes
E2	-1	top	3.52	no
E2	0	top	8.10	yes
E2	1	down	3.05	maybe
E2	2	down	4.01	maybe
E2	3	up	3.99	maybe
E2	4	up	3.85	yes
E2	5	down	2.32	maybe
E2	6	down	3.21	maybe
E2	7	up	2.44	yes
E2	8	up	3.10	no
E2	9	down	2.93	maybe
E2	10	down	3.36	yes
E2	11	up	1.81	no
E2	12	up	2.18	no
F1	0	top	2.66	maybe
F1	1	down	3.78	no
F1	2	down	3.78	maybe
F1	3	up	3.57	maybe
F1	4	up	5.59	yes
F1	5	down	3.30	maybe
F1	6	down	4.40	yes
F1	7	up	4.82	maybe
F1	8	up	2.70	yes
F1	9	down	2.61	maybe
F1	10	down	3.34	maybe
F1	11	up	4.59	yes
F1	12	up	3.21	no
F2	-1	top	4.08	no
F2	0	top	10.82	yes
F2	1	down	3.78	maybe
F2	2	down	3.52	maybe
F2	3	up	2.60	yes
F2	4	up	3.21	maybe
F2	5	down	2.86	no
F2	6	down	4.13	maybe
F2	7	up	3.33	maybe
F2	8	up	3.50	maybe
F2	9	down	3.78	maybe
F2	10	down	2.56	maybe
F2	11	up	2.32	no
F2	12	up	2.21	maybe
G1	0	top	2.64	no
G1	1	down	4.04	yes
G1	2	down	3.19	yes
G1	3	up	4.27	maybe
G1	4	up	2.13	no
G1	5	down	3.23	maybe
G1	6	down	3.38	maybe
G1	7	up	3.21	no
G1	8	up	4.08	yes
G1	9	down	2.81	maybe
G1	10	down	3.10	yes
G1	11	up	3.66	yes
G1	12	up	4.36	yes
G2	-1	top	6.27	maybe
G2	0	top	5.05	maybe
G2	1	down	3.38	maybe
G2	2	down	4.27	maybe
G2	3	up	4.06	yes
G2	4	up	3.90	yes
G2	5	down	3.00	maybe
G2	6	down	3.66	maybe
G2	7	up	3.66	no
G2	8	up	3.19	no
G2	9	down	2.63	maybe
G2	10	down	3.10	maybe
G2	11	up	3.64	no
G2	12	up	4.69	yes
H1	0	top	4.53	yes
H1	1	down	2.91	maybe
H1	2	down	2.32	no
H1	3	up	3.76	yes
H1	4	up	3.49	no
H1	5	down	3.74	yes
H1	6	down	2.81	maybe
H1	7	up	2.89	no
H1	8	up	2.98	yes
H1	9	down	3.06	no
H1	10	down	3.19	no
H1	11	up	2.70	yes
H1	12	up	2.91	maybe
H2	-1	top	7.06	yes
H2	0	top	5.94	maybe
H2	1	down	4.41	maybe
H2	2	down	4.22	maybe
H2	3	up	3.03	no
H2	4	up	5.42	yes
H2	5	down	4.32	maybe
H2	6	down	4.69	maybe
H2	7	up	3.54	no
H2	8	up	3.68	maybe
H2	9	down	3.38	maybe
H2	10	down	3.87	no
H2	11	up	3.71	maybe
H2	12	up	5.61	maybe
I1	0	top	4.87	yes
I1	1	down	2.49	maybe
I1	2	down	5.78	yes
I1	3	up	3.42	yes
I1	4	up	2.57	maybe
I1	5	down	1.96	maybe
I1	6	down	2.93	yes
I1	7	up	2.36	no
I1	8	up	2.23	yes
I1	9	down	2.00	maybe
I1	10	down	3.44	maybe
I1	11	up	3.51	yes
I1	12	up	2.72	no
I2	-1	top	6.90	maybe
I2	0	top	3.73	no
J1	0	top	2.27	no
J1	1	down	2.55	no
J1	2	down	3.38	maybe
J1	3	up	2.96	maybe
J1	4	up	2.79	yes
J1	5	down	3.15	maybe
J1	6	down	2.87	maybe
J1	7	up	3.21	maybe
J1	8	up	3.12	yes
J1	9	down	2.23	yes
J1	10	down	2.49	maybe
J1	11	up	2.78	no
J1	12	up	2.63	yes
K1	0	top	4.40	yes
K1	1	down	2.64	no
K1	2	down	2.59	maybe
K1	3	up	2.46	maybe
K1	4	up	2.63	yes
K1	5	down	2.89	maybe
K1	6	down	3.12	maybe
K1	7	up	3.12	no
K1	8	up	4.08	yes
K1	9	down	2.04	maybe
K1	10	down	2.32	yes
K1	11	up	3.21	no
K1	12	up	2.42	no
L1	0	top	3.76	yes
L1	1	down	2.76	maybe
L1	2	down	4.66	yes
L1	3	up	2.30	yes
L1	4	up	2.25	no
L1	5	down	2.85	maybe
L1	6	down	3.83	maybe
L1	7	up	2.23	maybe
L1	8	up	2.49	no
L1	9	down	2.15	yes
L1	10	down	2.79	maybe
L1	11	up	2.12	no
L1	12	up	2.98	no
M1	0	top	4.15	yes
M1	1	down	2.55	yes
M1	2	down	3.27	maybe
M1	3	up	1.81	no
M1	4	up	3.06	no
M1	5	down	2.89	maybe
M1	6	down	2.53	maybe
M1	7	up	3.74	maybe
M1	8	up	2.38	maybe
M1	9	down	2.34	no
M1	10	down	3.27	maybe
M1	11	up	2.81	yes
M1	12	up	2.63	no
N1	0	top	3.06	yes
N1	1	down	2.76	maybe
N1	2	down	2.89	maybe
N1	3	up	2.32	maybe
N1	4	up	2.06	no
N1	5	down	2.21	maybe
N1	6	down	2.98	maybe
N1	7	up	2.55	maybe
N1	8	up	5.02	yes
N1	9	down	2.38	maybe
N1	10	down	2.96	maybe
N1	11	up	3.13	maybe
N1	12	up	2.70	no

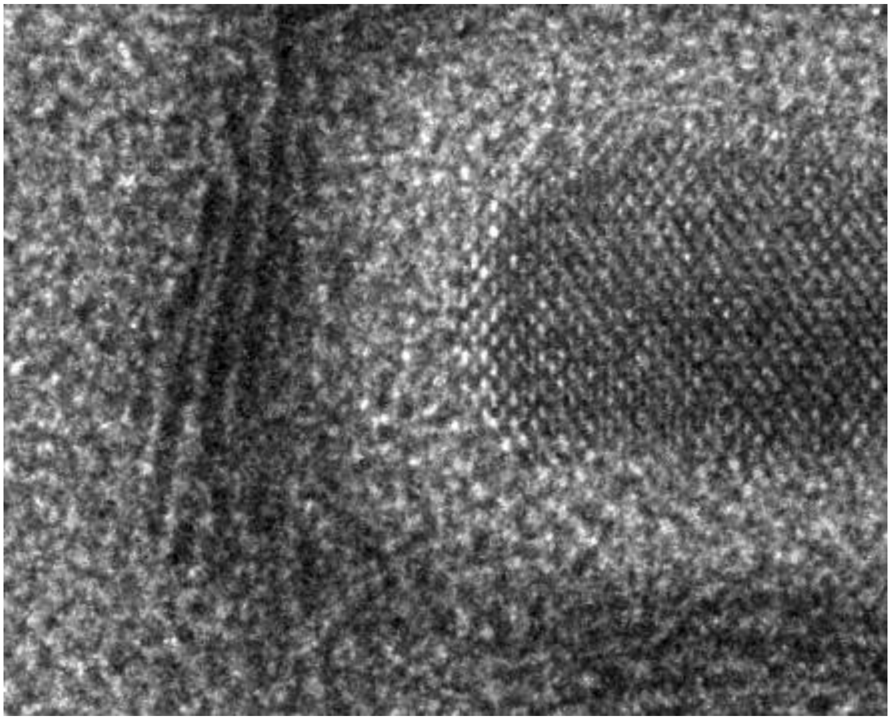

Figure: TEM image of an edge that was classified ‘undeterminable’.
